# Prognostic significance of 8-hydroxy-2′-deoxyguanosine in solid tumors: a meta-analysis

**DOI:** 10.1186/s12885-019-6189-9

**Published:** 2019-10-24

**Authors:** Xiangcheng Qing, Deyao Shi, Xiao Lv, Baichuan Wang, Songfeng Chen, Zengwu Shao

**Affiliations:** 1 Department of Orthopaedics, Union Hospital, Tongji Medical College, Huazhong University of Science and Technology, Wuhan, 430022 China; 2 Department of Orthopaedics, The First Affiliated Hospital of Zhengzhou University, Zhengzhou City, 450052 China

**Keywords:** 8-OHdG, Meta-analysis, Prognosis, Solid tumor, Reactive oxygen species, DNA oxidative damage

## Abstract

**Background:**

High level of reactive oxygen species (ROS) has been detected in almost all cancers, which make it become one of the best-characterized phenotypes in cancers. Though ROS plays an important role in tumors, the degree of oxidative stress can be better evaluated by assessing stable metabolites of oxidative reactions because of its high instability. 8-hydroxy-2′-deoxyguanosine (8-OHdG), a product of oxidative damage to 2′-deoxyguanosine, is known as a useful marker for assessing oxidative DNA damage and has been a feature of carcinogenesis in several researches. But the exact prognostic value of 8-OHdG expression in patients with cancer is still unclear.

**Methods:**

A comprehensive search was performed in PubMed, Web of Science, EMBASE. Eligible studies were included based on defined exclusion and inclusion criteria to perform a meta-analysis. STATA 14.0 was used to estimate pooled hazard ratios (HRs) with 95% confidence interval (95% CI), the heterogeneity among studies and publication bias to judge the prognostic value.

**Results:**

A total of 2121 patients from 21 eligible studies were included in the meta-analysis. A significant association was found between elevated 8-OHdG expression and poor OS (overall survival) in cancer patients (pooled HR 1.921, 95% CI: 1.437–2.570); In the subgroup analysis, race of sample, cancer types, detection method of 8-OHdG, sample classification, detection location of 8-OHdG and paper quality (score more or less than 7) did not alter the association between 8-OHdG expression and cancer prognosis. Furthermore, 8-OHdG expression was an independent prognostic marker for overall survival in patients with cancer (pooled HR 2.110, 95% CI: 1.482–3.005) using Cox multivariate analyses.

**Conclusions:**

This meta-analysis found that highly expressed 8-OHdG in tumor tissues may be a predictor of prognosis in most solid tumors. However, especially in breast cancer, low 8-OHdG expression is associated with poor prognosis, which is partly because of the increased antioxidant mechanisms in breast cancer tissues. This study demonstrates for the first time that 8-OHdG expression is associated with the prognosis of cancer patients. In the future, whether the expression level of 8-OHdG can be used as a biomarker for the prognosis of all human cancers requires more research.

## Background

Tumor cells constantly suffer various endogenous and environmental attacks, which make high level of reactive oxygen species (ROS) be detected in almost all cancers and become one of the best-characterized phenotypes [1–3]. The role of ROS in cancer is a “doubled edged sword”. ROS can serve as a carcinogenic factor through promoting tumorigenesis, development and spread of cancers by activating or regulating signaling pathways that affect tumor cell survival, proliferation and metastasis [4–6]. However, high levels of ROS can also play a role in tumor suppression by inhibiting cell proliferation and inducing cell death [7–9]. Many cancer treatments, such as radiotherapy and certain chemotherapy agents, act through oxidative stress pathways via the production of ROS to suppress tumor growth and progression [10]. In order to prevent cell death, cancer cells can scavenge reactive oxygen species to adapt high levels of ROS and activate pro-tumorigenic signaling pathways, by upregulating antioxidant pathways and regulatory factors [11–13].

Though ROS plays an important role in tumors, the degree of oxidative stress can be better evaluated by assessing stable metabolites of oxidative reactions because of its high instability. ROS can cause oxidative damage to double-stranded DNA directly, or to free bases in the cellular and mitochondrial deoxynucleoside triphosphate (dNTP) pool [14]. Among all the nucleobases, guanine is the most susceptible to oxidation by ROS [15]. Oxidative damage to 2′-deoxyguanosine produces 8-hydroxy-2′-deoxyguanosine (8-OHdG). The formation of 8-OHdG on DNA can cause G:C—T:A mispairing mutations, which are considered to have a close relationship with the development and progression of tumors, cell ageing and some degenerative diseases [16].

There is an increasing body of evidence indicating that 8-OHdG is a useful marker for assessing oxidative DNA damage and has been a feature of carcinogenesis in several researches [17, 18]. High levels of 8-OHdG in tumors, blood samples or urine have been found in various cancers and implicated as a promising marker for predicting the prognosis of cancers [19–40]. However, the association of oxidative damage to DNA with tumors still needs to be more extensively investigated and most studies reported so far are limited in discrete outcome and sample size. For these reasons we performed a quantitative meta-analysis and systematic review to gain better insight into the prognostic value of 8-OHdG expression in patients with cancer.

## Methods

### Search strategy

This analysis was conducted following the meta-analyses and systematic reviews guidelines for prognosis-related tumor marker researches [41, 42]. An electronic search of PubMed, Web of Science, EMBASE was performed independently by two authors (XQ and DS) prior to May 15, 2018. Search terms were used in all possible combinations as following: 7,8-dihydro-8-oxodeoxyguanosine, 8-hydroxy-2′-deoxyguanosine, 8-hydroxy-2′- deoxyguanosine, 8-OHdG, 8OHdG, 8-OH-dG, 8-OHG, 8-oxo-G, 8-oxo-dG, 8-hydroxydeoxyguanosine, 8-oxo-guanine, 8-hydroxyguanine, 8-hydroxyguanosine, 8-oxo-2-deoxy guanosine, 8-oxo-7,8-dihydro-2-deoxyguanosine, 8-oxo-7,8-dihydro- 2′-deoxyguanosine, 8-hydroxy-2-deoxyguanosine, 8-oxo-7,8-dihydro-2-deoxyguanosine, tumor, cancer, sarcoma, carcinoma, neoplasm, malignancy, prognosis, mortality of metastasis, progression, development, outcome, survival, recurrence, clinical significance. Conflicts were solved through group discussion.

### Inclusion and exclusion criteria

Studies included in the present meta-analysis were independently reviewed by two investigators (XQ and DS) and should meet the following criteria: (1) The prognostic data of 8-OHdG in any type of human solid tumors needed to be presented; (2) All cancer patients were diagnosed according to the gold standard for diagnosis, based on histopathological examinations; (3) 8-OHdG levels in tumors, blood samples or urine were estimated in each study; (4) The patients were divided into two groups according to the levels of 8-OHdG; (5) Sufficient data should be provided to obtain hazard ratios (HR) for survival rates and their 95% confidence intervals (95%CI). Studies were excluded from the present meta-analysis if one of the following criteria was met: (1) Case reports, reviews, meta-analysis, letters, editorials, comments, expert opinions or any other reviews that didn’t contain raw data; (2) Full text could not be obtained; (3) Researches on non-English writing; (4) Repetitive publications; (5) No survival data or data insufficient to be extracted and analyzed; (6) Survival data was acquired based on animal studies and no follow-up of patients. Detailed inclusion and exclusion criteria of each study are presented in Additional file 1: Table S1.

### Data extraction and quality assessment

Data was extracted independently by the two researchers (XQ and DS), and final consensus was reached through discussion. Data were retrieved from each study including: author; year of publication; country of the population enrolled; ethnicity; tumor stage; sample size; study design; follow-up data; survival data; survival analysis methodology; expression levels, location and laboratory methods of 8-OHdG; cut-off values; HR values and their 95% confidence intervals. Quality assessment of cohort studies in this meta-analysis was performed using the Newcastle-Ottawa scale (NOS) as recommended by the Cochrane Non-Randomized Studies Methods Working Group. Studies with score ≥ 7 were considered high quality according to the NOS. Detailed NOS scores of all included studies were shown in Table 1.

**Table 1:** Characteristics of studies included in the meta-analysis

Author	Region	Cancer Type	Sample size	Tumor stage	Follow-up (month)	Outcome measure	Expression associates with poor prognosis	Assay	Cut-off value	Location of 8-oxo-dG	Survival analysis	NOS score	Method*
Li et al. 2012 [22]	China	Hepatocellular carcinoma	103	I-IV	36	OS	High	IHC	percentage of positive tumor cells	Nuclei	multivariate	6	1
Karihtala et al. 2009 [19]	Finland	Ovarian cancer	68	I-IV	41	OS	High	IHC	median	Nuclei	multivariate	6	1
Ma-on et al. 2017 [23]	Thailand	Hepatocellular carcinoma	53	NA	80	OS	High	IHC	IHC score 12	Nuclei	NA	5	2
Xu et al. 2013 [24]	China	Ovarian cancer	72	I-IV	Over 120	OS, PFS	High	ELISA	Fold change	NA	univariate, multivariate	8	1,2
He et al. 2014 [25]	China	Esophageal cancer	144	I-IV	60	OS	High	IHC	percentage of positive tumor cells	Nuclei	multivariate	8	1,2
Shen et al. 2007 [26]	USA	Nonsmall-Cell Lung cancer	99	I-IV	82	OS	High	ELISA	median	NA	multivariate	7	1
Soini et al. 2011 [27]	Finland	Bladder carcinoma	252	I-IV	300	OS	High	IHC	positive > 5%	Nuclei	NA	6	2
Dziaman et al. 2014 [20]	Poland	Colorectal cancer	79	I-IV	100	OS	High	LCEC	median	NA	NA	6	2
Jakovcevic et al. 2015 [21]	Croatia	Breast cancer	145	I-IV	112	OS, DFS	Low	IHC	percentage of positive tumor cells	Nuclei	univariate multivariate	6	1
Pylväs et al. 2011 [29]	Finland	Ovarian cancer	84	I-IV	Over 125	OS	High	IHC, ELISA	percentage of positive tumor cells for IHC. 140 pg/mL for ELISA	NA	univariate multivariate	6	2
Aman et al. 2017 [30]	Japan	Ovarian cancer	95	I-IV	208	OS	High	IHC	percentage of positive tumor cells	Nuclei	univariate	6	1
Matosevic et al. 2015 [31]	Croatia	Colorectal cancer	138	I-IV	169	OS	High	IHC	percentage of positive tumor cells	Cytoplasm	multivariate	7	1
Matsumoto et al. 2003 [32]	Japan	Hepatocellular carcinoma	73	NA	Over 60	CSS, RFS	High	IHC	percentage of positive tumor cells	NA	univariate multivariate	8	1,2
Hintsala et al. 2016 [33]	Finland	Melanoma	121	NA	Over 150	CSS	Low	IHC	NA	Nuclei	multivariate	6	1
Murtas et al. 2010 [34]	Italy	Melanoma	46	I-II	60	OS	High	IHC	percentage of positive tumor cells	Nuclei	multivariate	8	1
Sheridan et al. 2009 [35]	Ireland	Colorectal cancer	113	I-IV	80	OS	High	IHC	NA	Nuclei	multivariate	6	1
Karihtala et al. 2011 [36]	Finland	Breast cancer	79	I-III	60	CSS	Low	IHC	NA	Nuclei	univariate multivariate	6	2
Maki et al. 2007 [37]	USA	Hepatocellular carcinoma	30	I-II	NA	DFS	High	IHC	percentage of positive tumor cells	NA	multivariate	6	1
Pylväs-Eerola et al. 2015 [38]	Finland	Ovarian cancer	105	I-IV	NA	OS, DFS	High	ELISA	median	NA	multivariate	6	1,2
Miyake et al.2004 [39]	Japan	Renal cell carcinoma	72	I-IV	NA	CSS	High	ELISA	mean plus one standard deviation	NA	multivariate	6	1
Sova et al. 2010 [40]	Finland	Breast cancer	150	I-IV	NA	CSS	Low	IHC	percentage of positive tumor cells	Nuclei	multivariate	6	1

*OS* overall survival, *DFS* disease free survival, *PFS* progression free survival, *RFS* recurrence free survival, *CSS* cancer specific survival, *NOS* Newcastle-Ottawa Scale, *IHC* Immunohistochemistry, *ELISA* Enzyme-linked immunosorbent assay, *LCEC* Liquid chromatography electrochemistry, *NA* not available

*1 denoted as obtaining HRs directly from publications; 2 denoted as HRs were extracted and calculated from Kaplan-Meier curves

### Statistical analysis

The meta-analysis was performed as previously described [43]. In the present study, statistical analysis and graphical representation were performed using Stata version 14.0 (Stata Corporation, College Station, TX, USA). Pooled HRs and ORs with 95%CIs were used to evaluate the association between 8-OHdG expression and prognosis. HRs or ORs with 95%CIs can be directly obtained from most included studies or estimated from the existing data using methods as previously described [41]. An HR > 1 indicates a worse outcome of patient with high 8-OHdG expression, while an HR < 1 implied a worse survival for patients with decreased 8-OHdG expression. The test for heterogeneity of combined HRs was carried out using a χ^2^ based Cochran Q test and Higgins I^2^ statistic. I^2^ values > 50% indicated heterogeneity among studies. If there existed heterogeneity, a random-effect model, subgroup analysis and meta regression by factors contributing to heterogeneity would be carried out. Influence analyses was performed to examine the effect of each study on the overall pooled results. The presence of publication bias was evaluated by using funnel plots, Begg’s test and Egger’s test. *P* values < 0.05 were considered statistically significant.

## Results

### Included studies and characteristics

**Fig. 1:**
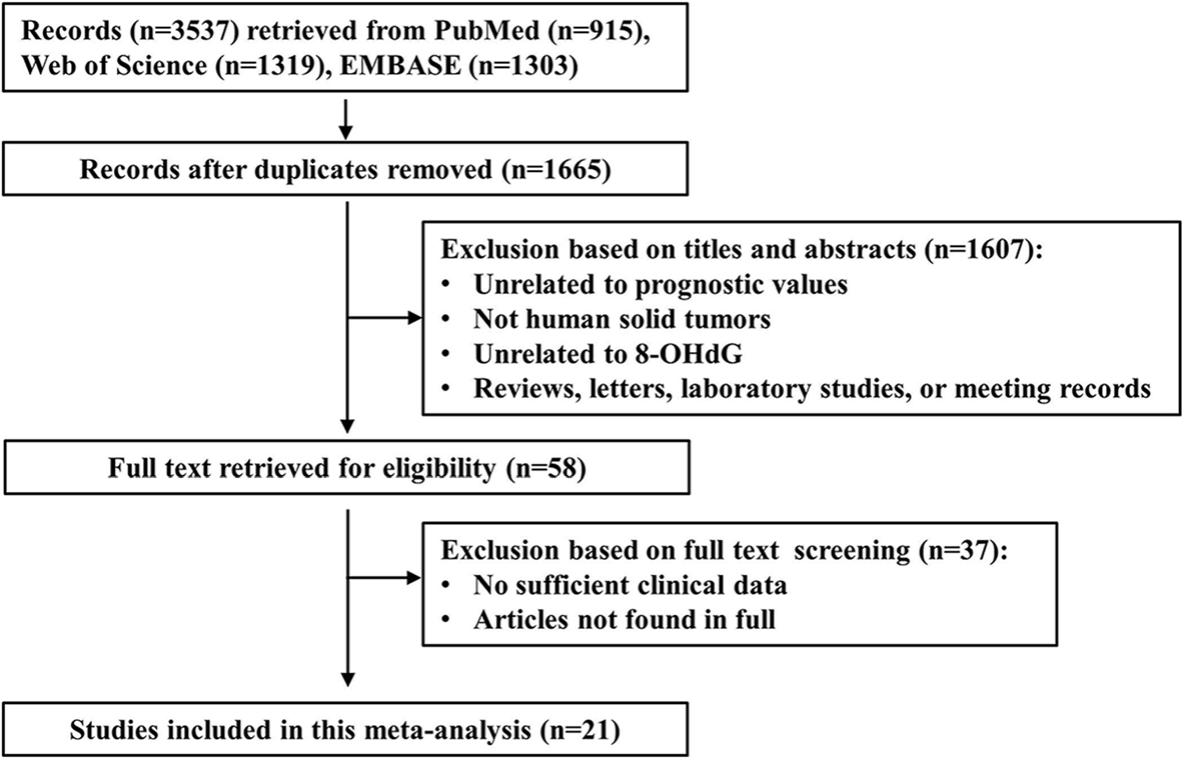
The flow diagram of the meta analysis

Based on our searching strategy, a total of 3537 articles were identified from PubMed (*n* = 915), Web of Science (*n* = 1319) and EMBASE (*n* = 1303). After removing duplicates, 1665 articles were left. Furthermore, 1607 of the remaining articles were excluded according to the titles and abstracts. Finally, a total of 21 relevant articles were included in this meta-analysis after a more careful full-text reading. The detailed screening process is shown in Fig. 1.

Among the 21 studies, a total of 2121 patients were included, with mean sample size of 101 patients (range 30 to 252). The period of these studies ranged from 2003 to 2017. The regions represented in the studies include various countries around Europe, Asia and America, of which the race contains both Caucasoid and Mongoloid. Eight different types of cancer were evaluated. Most studies analyzed the expression level of 8-OHdG by IHC or ELISA, while there was one study unitizing liquid chromatography electrochemistry. Overall survival (OS), cancer-specific survival (CSS), recurrence-free survival (RFS), disease-free survival (DFS) and progression-free survival (PFS) were estimated as survival outcomes in the studies. RFS, DFS and PFS were merged into the event-free survival (EFS) group for analysis. Cox multivariable analyses were performed in 17 studies. Further detailed characteristics of each study are presented in Table 1.

**Fig. 2:**
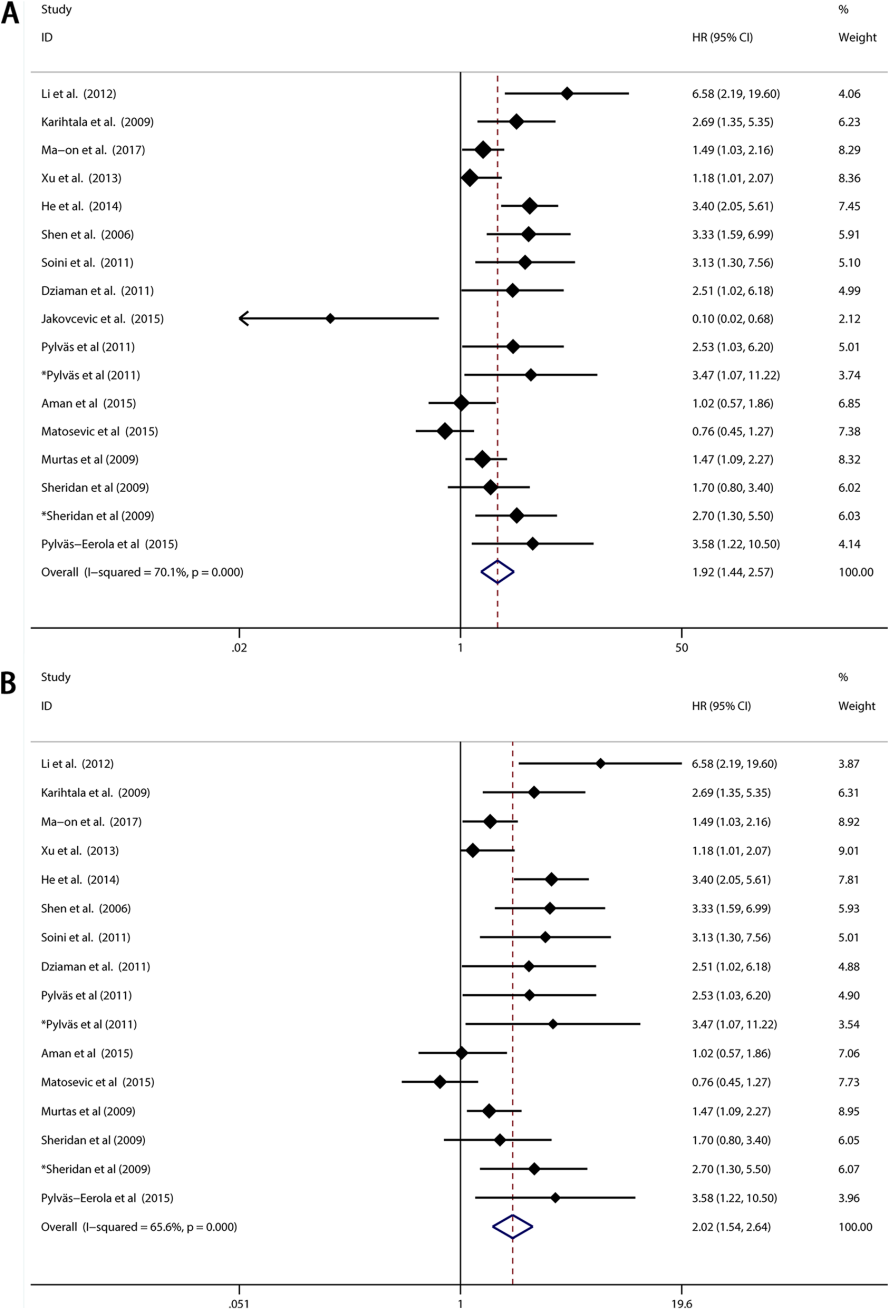
Meta-analysis of the pooled HRs of OS with elevated 8-OHdG expression in cancer patients. **a** All studies included. **b** Study of Jakovcevic et al. excluded

**Fig. 3:**
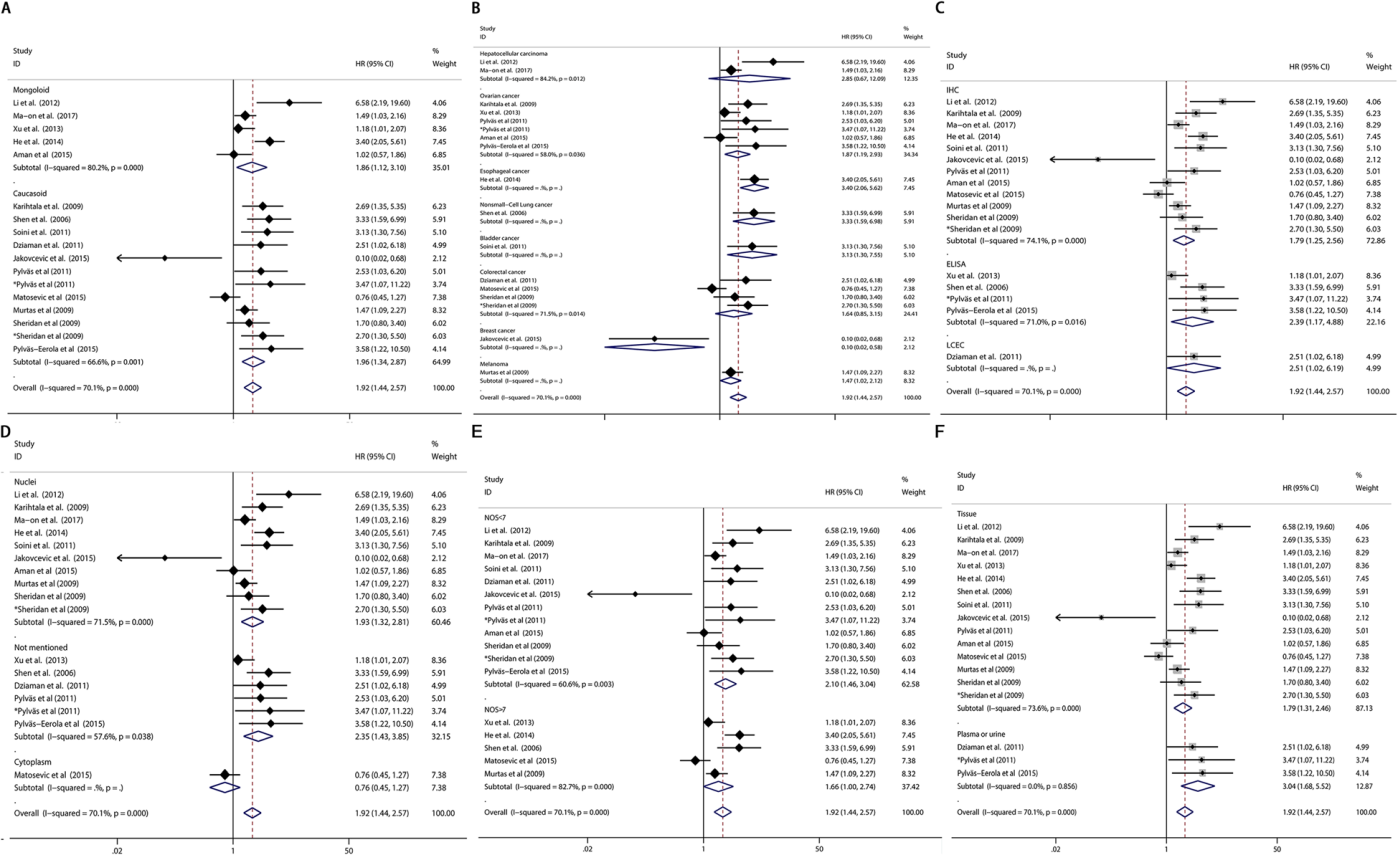
Subgroup analysis of the pooled HRs of OS by various factors. **a** Subgroup analysis of HRs of OS by factor of race. **b** Subgroup analysis of HRs of OS by factor of cancer types. **c** Subgroup analysis of HRs of OS by factor of detection method of 8-OHdG. **d** Subgroup analysis of HRs of OS by factor of detection location of 8-OHdG. **e** Subgroup analysis of HRs of OS by factor of research quality. **f** Subgroup analysis of HRs of OS by factor of sample classification

Overall survival (OS) based on different 8-OHdG expression levels was reported in 8 types of solid tumors from 15 of the 21 included studies with a total of 1596 patients. Elevated 8-OHdG was significantly associated with poor OS in these patients (pooled HR 1.921, 95%CI: 1.437–2.570) (Fig. 2a), while significant heterogeneity was found in these studies (Tau^2^ = 0.2298; χ^2^ = 53.52, df = 16, *p* < 0.0001; I^2^ = 70.1%). Since obvious heterogeneity was observed, subgroups analysis was performed by factors of the race of sample, cancer types, detection method of 8-OHdG, detection location of 8-OHdG, sample classification and research quality (Fig. 3). Detailed results of subgroup analysis were demonstrated in Table 2. Despite the subgroup of hepatocellular carcinoma (Cancer Types) and the subgroup of cytoplasm (Detection location of 8-OHdG), the significant association between 8-OHdG expression and poor OS could be observed in each subgroup. We further performed meta-regression with the covariates including above factors to explore the source of heterogeneity. From the result we found that p<0.05 was only observed in the subgroup of breast cancer (Cancer types) covariate, which implied that the subgroup of breast cancer may be the major source of heterogeneity. The study of Jakovcevic et al. enrolled patients with breast cancer and drew a conclusion that negative 8-OHdG expression was a poor prognostic biomarker, which was contrary to the other researches. It could be a consequence caused by cancer specificity. We discussed this point in the discussion part below.

**Table 2:** Subgroup analysis of pooled HR of OS by various factors with elevated 8-OHdG expression

Subgroup analysis	No. of studies	No. of patients	Pooled HR (95%CI)	Meta regression (*p* -value)	Heterogeneity	
					I^2^	*p* -value
Race						
Caucasoid	12	1129	1.962 [1.341–2.870]	0.907	66.6%	0.001
Mongoloid	5	467	1.862 [1.117–3.104]	–	80.2%	< 0.001
Cancer types						
Hepatocellular carcinoma	2	156	2.853 [0.673–12.089]	0.727	84.2%	0.012
Ovarian carcinoma	6	424	1.867 [1.190–2.930]	0.464	58.0%	0.036
Colorectal cancer	4	330	1.637 [0.850–3.153]	0.352	71.5%	0.014
Esophageal cancer	1	144	3.400 [2.055–5.624]	0.982	–	–
Nonsmall-Cell Lung cancer	1	99	3.330 [1.588–6.982]	–	–	–
Melanoma	1	46	1.470 [1.019–2.121]	0.367	–	–
Breast cancer	1	145	0.100 [0.017–0.583]	0.019	–	–
Bladder cancer	1	252	3.130 [1.298–7.548]	0.950	–	–
Detection method of 8-OHdG						
IHC	12	1157	1.787 [1.246–2.563]	0.646	74.1%	< 0.001
ELISA	4	360	2.386 [1.167–4.881]	0.947	71.0%	0.016
LCEC	1	79	2.510 [1.018–6.187]	–	–	
Sample classification						
Tissue	14	1412	1.792 [1.307–2.458]	–	73.6%	< 0.001
Plasma or urine	3	268	3.042 [1.676–5.519]	0.006	0.0%	0.856
Detection location of 8-OHdG						
Nuclei	10	1019	1.927 [1.321–2.810]	0.596	71.5%	< 0.001
Cytoplasm	1	138	0.759 [0.454–1.268]	0.118	–	–
Not mentioned	6	439	2.345 [1.429–3.848]	–	57.6%	0.038
research quality						
NOS score ≥ 7	5	499	1.658 [1.002–2.743]	0.526	82.7%	< 0.001
NOS score < 7	12	1097	2.104 [1.456–3.040]	–	60.6%	0.003

**Fig. 4:**
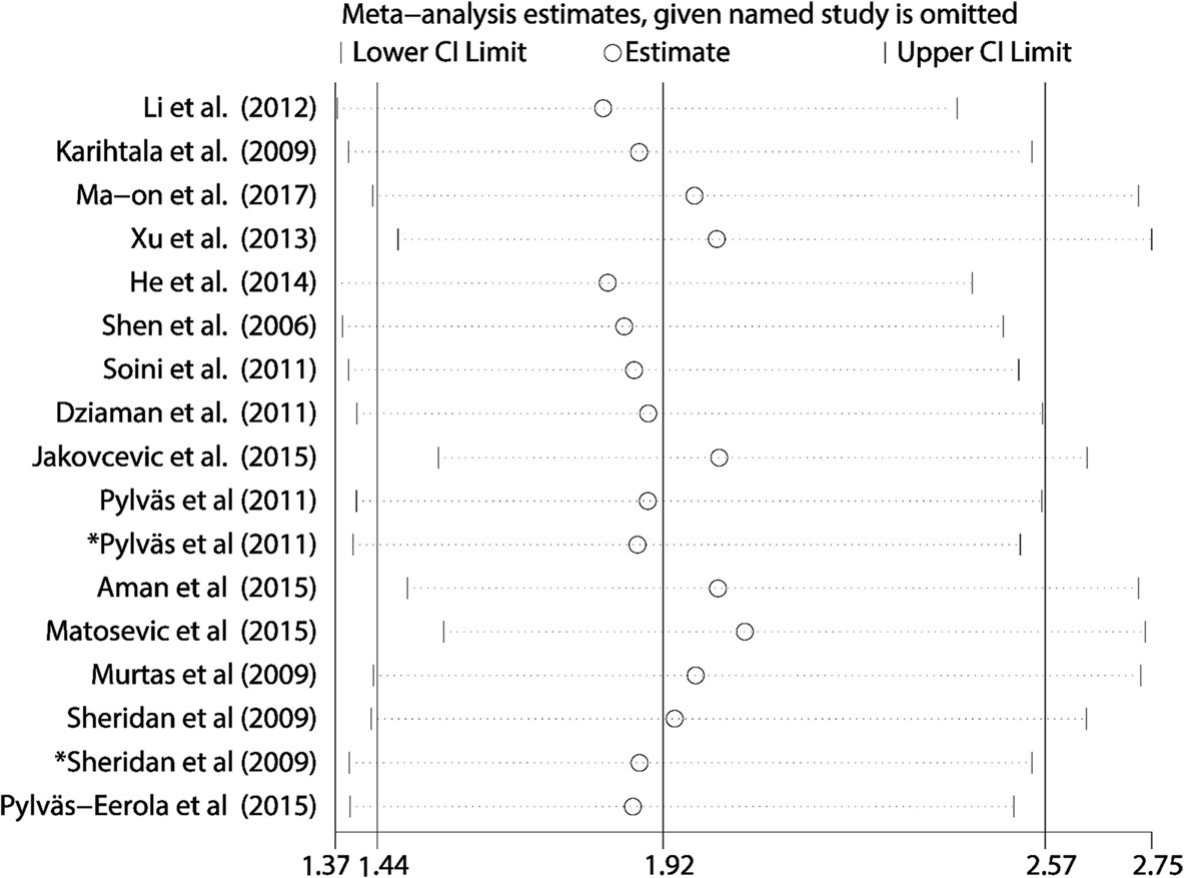
Influence analysis of the included studies for OS. No obvious change of the pooled HRs and 95% confidence intervals was observed after excluding any included study

**Fig. 5:**
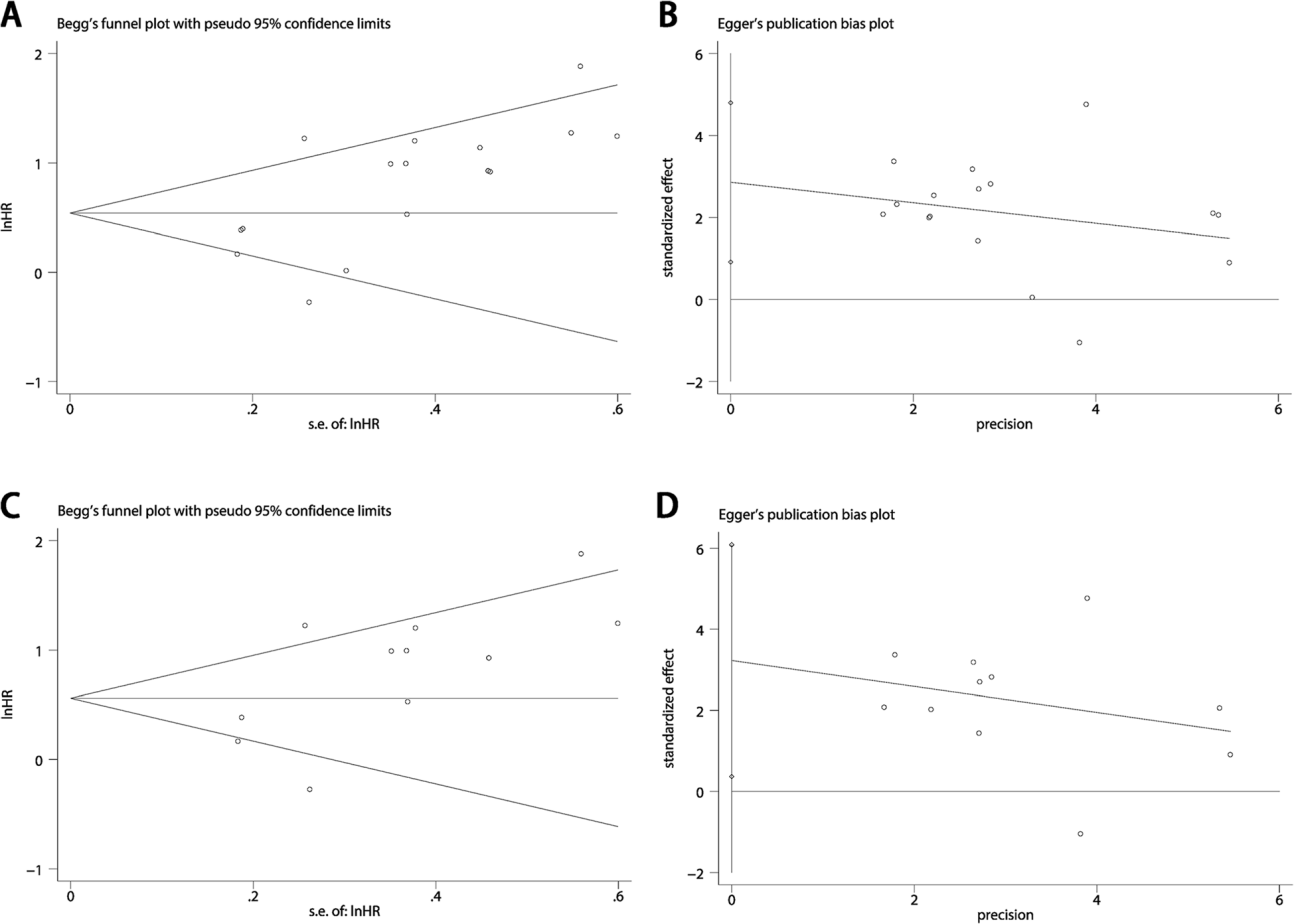
Plot of publication bias analysis. **a** Begg’s test and (**b**) Egger’s test for analysis of the association between 8-OHdG expression and OS. **c** Begg’s test and (**d**) Egger’s test graph for analysis of the independent role of 8-OHdG expression for OS

**Fig. 6:**
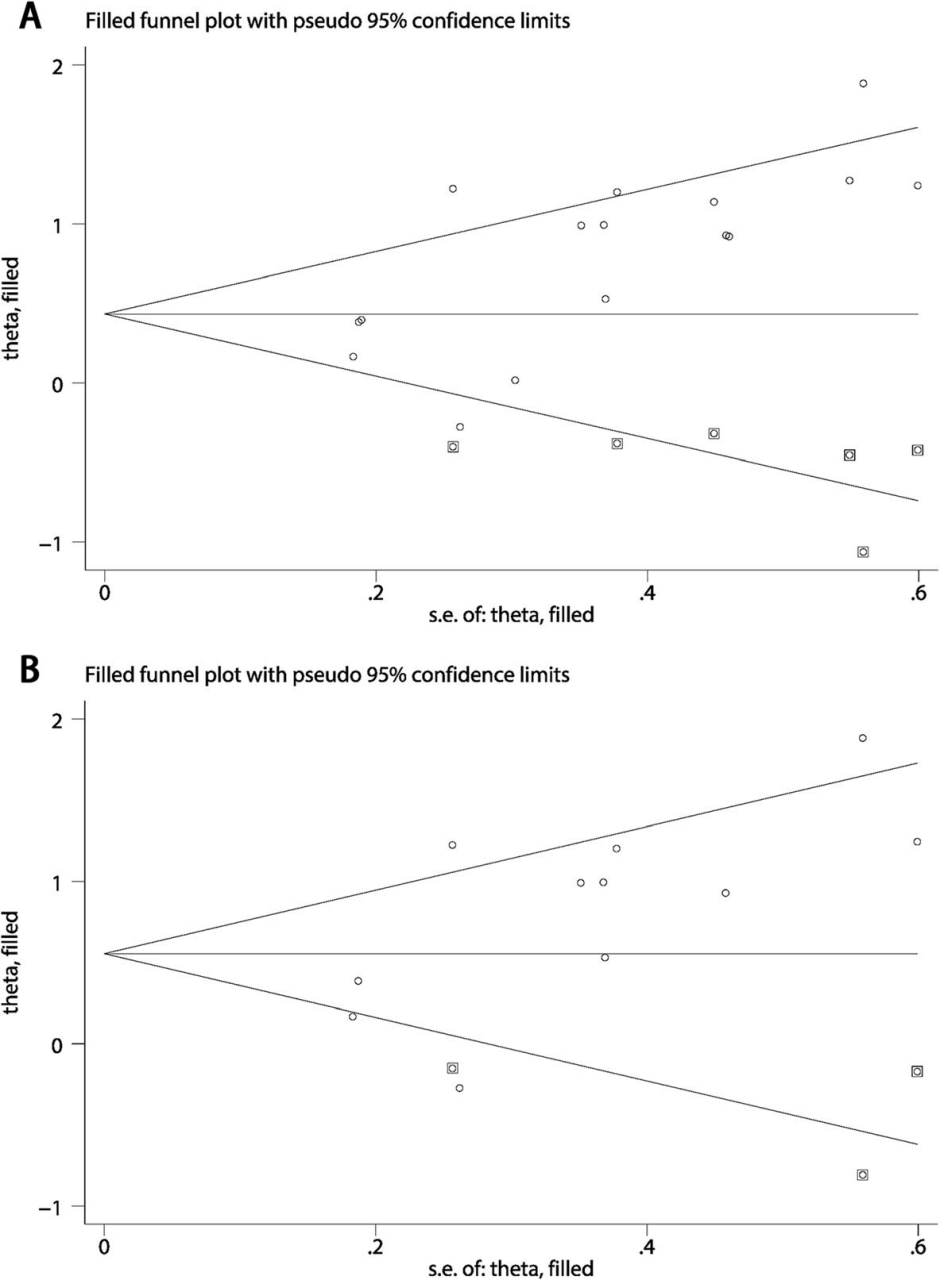
Plot of the “Trim and fill” analysis. **a** Analysis of the association between 8-OHdG expression and OS. **b** Analysis of the independent role of 8-OHdG expression for OS

Base on the above result of meta-regression, we excluded the study of Jakovcevic et al. and still found significant association between elevated 8-OHdG expression and poor OS in cancer patients (pooled HR 2.022, 95% CI: 1.540–2.641) with reduced heterogeneity (I^2^ = 65.5%) (Fig. 2b). Furthermore, as shown in Fig. 4, influence analysis was carried out for purpose of ensuring the stability of the result. No obvious change of the pooled HR and 95% CIs could be observed after excluding any study from the whole studies. In aspect of the publication bias, Begg’s test and Egger’s linear regression test were performed. The Begg’s tests proved that there was no evidence of publication bias (*p* = 0.053) while the Egger’s test showed there was significant publication bias (*p* = 0.007) (Fig. 5a and Fig. 5b). Thus “Trim and fill” analysis was conducted and the result estimated that 8 studies evaluating the association between expression of 8-OHdG and overall survival of cancer patients were remaining unpublished. The result of filled meta-analysis was pooled HR 1.545, 95% CI: 1.179–2.026, which exhibited that the significant association between elevated 8-OHdG expression and poor OS in cancer patients maintained unchanged (Fig. 6a).

**Fig. 7:**
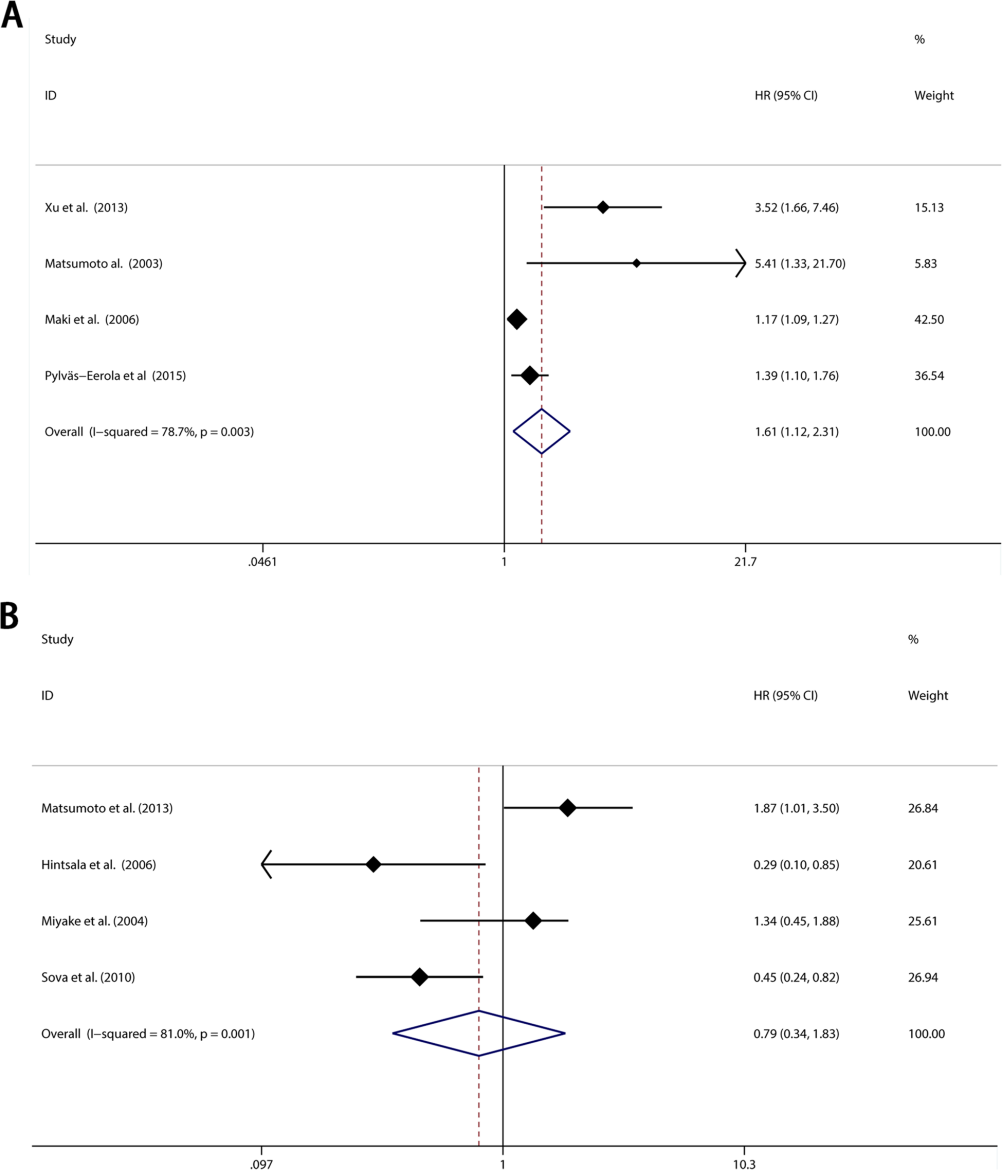
**a** Meta-analysis of the pooled HRs of EFS with elevated 8-OHdG expression in cancer patients. **b** meta-analysis of the pooled HRs of CSS with elevated 8-OHdG expression in cancer patients

Among the 21 included studies, four studies reported event-free survival (EFS) in 489 patients. A close relationship was observed between elevated 8-OHdG expression and EFS (pooled HR 1.612, 95% CI: 1.121–2.310, I^2^ = 78.7%) (Fig. 7a). However, due to the limited number of included studies, appraisal of publication bias was not performed.

There were 5 studies reported the association between 8-OHdG expression and cancer-specific survival (CSS), corresponding to hepatocellular carcinoma, melanoma, renal cell carcinoma and breast cancer, including a total of 495 patients. After summarizing the results, we found there was no significant association between 8-OHdG expression and CSS (pooled HR 0.793, 95%CI: 0.344–1.828, I^2^ = 81.0%) (Fig. 7b). We need to point out that this result is contrasted to the other results above.

**Fig. 8:**
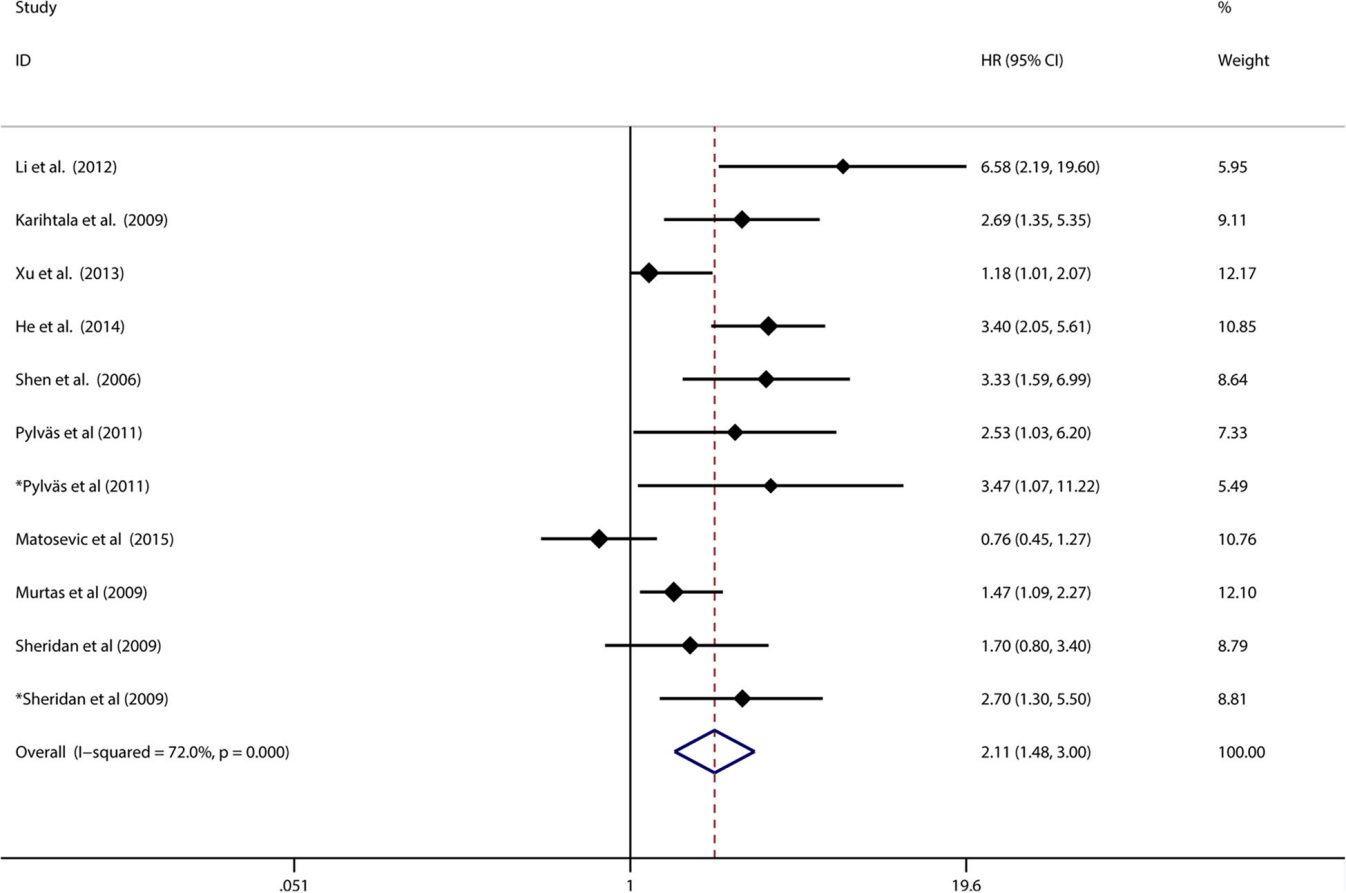
Meta-analysis of the independent role of elevated 8-OHdG in OS in cancer patients

A total of 11 studies including 1243 patients used Cox multivariate analysis to assess whether 8-OHdG expression could be an independent prognostic factor for OS of cancer patients. Elevated 8-OHdG as an independent factor for poor prognosis was found alone in nine of them. The results of Cox multivariate analyses in these 11 studies showed that 8-OHdG expression was an independent prognostic factor for overall survival (pooled HR 2.110, 95% CI:1.482–3.005), and heterogeneity was still observed among studies (Tau^2^ = 0.2339; χ^2^ = 35.73, df = 10, *p* < 0.0001; I^2^ = 72.0%). (Fig. 8).

As for the publication bias, the Begg’s test (*p* = 0.276) and Egger’s test (*p* = 0.031) showed opposite conclusion. (Fig. 5c and Fig. 5d) Thus we applied the “Trim and fill” analysis to confirm our result. There were 3 studies evaluating whether 8-OHdG expression could be an independent prognostic factor for OS remaining unpublished. The result of filled meta-analysis was pooled HR 1.793, 95% CI: 1.242–2.436, which confirmed that elevated 8-OHdG could be an independent factor for poor prognosis of overall survival after the “Trim and fill” analysis. (Fig. 6b).

## Discussion

Cancer is a major public health problem worldwide and is the second leading cause of death in the United States [44]. The 5-year survival of many cancers is still quite low. For most types of cancers, the pathological staging is a gold standard to predict its prognosis. However, patients with the same tumor stage often exhibit quite different clinical outcomes, which suggests that this conventional method is unable to precisely predict the prognosis of cancer patients. Therefore, new potential biomarkers for prognosis and diagnosis are urgently needed to improve the prognosis of cancer patients.

From the important role of oxidative stress in cancer treatment, progression and metastasis, we infer that it may also be particularly important in cancer prognosis. However, ROS is so instable that it’s not easy to be precisely detected and the degree of oxidative stress can be better assessed by detecting its stable metabolites. 8-OHdG, a typical biomarker of oxidative stress, can originate from 8-oxo-dGTP in the nucleotide pool, or by direct oxidation of guanine base in DNA. MTH1 (MutT Homolog 1) with 8-oxo-dGTP hydrolyzing activity, OGG1 (8-oxoguanine DNA glycosylas) with 8-OHdG DNA glycosylase activity and MUTYH (MutY homolog) with adenine DNA glycosylase activity, all play roles in minimizing 8-oxoG accumulation in cellular DNAs [45]. Thus, the levels of 8-OHdG measured in tumor tissues may be representative of the DNA oxidative damage-repair ability of the cell and an intermediate biomarker of the extent of accumulated intratumoral oxidative DNA damage [26]. High levels of 8-OHdG in tumors, blood samples or urine have been found in various cancers and implicated as a promising marker for predicting the prognosis of cancers [19–40]. Nevertheless, the exact relationship between DNA oxidative damages and tumors is still unknown. To the best of our knowledge, this is the first meta-analysis performed to obtain a comprehensive insight into the prognostic value of 8-OHdG in solid tumors.

In our meta-analysis, we examined 21 independent studies enrolling a total of 2121 cancer patients. After systematic review of these studies, we discovered that 8-OHdG was highly expressed in various types of tumors except a few specific tumors such as breast cancer. By combining the survival data obtained from these studies, we found that high 8-OHdG expression was a biomarker for poor prognosis for overall survival in most solid cancer patients.

Because obvious heterogeneity was observed among studies, we performed s subgroup analysis, meta regression analysis and influence analysis to examine the source of heterogeneity and the stability of the pooled result. In subgroup analysis, we still found that high 8-OHdG expression was associated with poor overall survival in most subgroups. The factors such as race of sample, cancer types, detection method of 8-OHdG, detection location of 8-OhdG, sample classification and research quality would not influence the pooled result. Meta regression analysis found that the subgroup of breast cancer would be the major source of heterogeneity. After excluding the corresponding study, we could still find significant association between elevated 8-OHdG expression and poor OS in cancer patients with reduced heterogeneity. In addition, influence analysis was performed and confirmed the stability of our pooled result. Furthermore, through summarizing the data from studies using Cox multivariate analysis, we found that 8-OHdG could be an independent prognostic risk factor for overall survival. Besides, by collecting the survival data of cancer recurrence or progression, we found that elevated 8-OHdG expression was associated with event-free survival of cancer patients. However, the number of these studies was relatively limited, which made the conclusion not so convincing as above. It should be noted that there were three studies reporting the association between 8-OHdG expression and prognosis of breast cancer patients. One was analyzed with overall survival data and the other two were cancer specific survival data. All of the three studies reported that negative or weak 8-OHdG expression was associated with poor survival of breast cancer patients. These results were contrasted with the other studies and the pooled result.

There are several potential mechanisms behind the different association of 8-OHdG levels and tumor prognosis in breast cancer. To deal with the threats posed by high ROS production, tumor cells evolve lots of antioxidant mechanisms, which would prevent ROS from interacting with DNA or directly eliminate 8-OHdG, thus decreasing the expression level of 8-OHdG in tumor tissues. For example, transcription factor NF-E2-related factor 2 (Nrf2), the main inductor of multiple antioxidant enzymes, has been revealed to be highly expressed in various cancer cells [33, 46–49]. Nrf2 up-regulation and consequent antioxidant enzyme induction may lead to low expression level of 8-OHdG and counteract the negative effect of ROS, which would promote cancers progression and potentially metastasis. This may explain why patients with low 8-oxodG levels have worse prognosis in breast cancer patients [36, 40]. This mechanism was also demonstrated in melanoma [33].

In our study, a few limitations should be pointed out. First, the cut-off values of high and low 8-OHdG expression were different among studies. Most were set to be the median, while some of them were set by different standards. Second, as for the race of included patients, there were only Caucasoid and Mongoloid, the representativeness of our results could be limited. Third, several HRs could not be directly obtained from the publications. Data extracted and calculated through survival curves might not be precise enough. Fourth, the association between 8-OHdG expression and clinicopathological characteristics could not be analyzed due to the insufficient data. Therefore, larger-scale, multicenter, and high-quality studies are highly necessary to further confirm our findings. Fifth, although we have confirmed that all the antibodies used in involved studies were mouse original and commercial antibodies, it’s definite that different clones may target different parts of the interest protein, which may possibly be a source of heterogeneity. Furthermore, it is necessary to discuss those different samples with various detecting laboratory methods to evaluate 8-OHdG. Because there hasn’t been a golden standard technique for detecting 8-OHdG, different samples (shown in Table 1) were used in the included studies. Although high-pressure liquid chromatography measurements are preferred by some investigators, it is a technically difficult method, takes a long time, and has some limitations (further 8-OHdG lesions can be artificially produced during DNA extraction and sample preparation) [50]. Excretion of 8-OHdG with urine represents the average rate of oxidative stress/DNA damage in the whole body. High urinary levels of oxidized DNA-derived metabolites have been reported in several pathological conditions [51], which indicate that it can not precisely represent the exact levels of 8-OHdG and DNA oxidative damages in tumor tissues. These might represent a potential source of heterogeneity. However, subgroup analysis and meta-regression using different laboratory methods with different biological samples (cancerous tissues, plasma or urine) for the measurement of 8-OHdG showed they were not the major source of heterogeneity. Another potential reason why obvious heterogeneity was observed in the current meta-analysis may be partially due to the different locations of 8-OHdG detected in the included studies. 8-OHdG is a major product of ROS damages to DNA and mainly located in nuclei. In order to localize the 8-OHdG, most included studies analyzed the expression levels of 8-OHdG using immunohistochemical method. However, there are also some limitations in immunohistochemistry, such as it can be only used as a method of semi-quantitative analysis and results in different studies are evaluated according to different standards and cut-off values. Nevertheless, in consistent with different biological samples, subgroup analysis and meta-regression in different locations of 8-OHdG (nuclei, cytoplasm or not mentioned) for the measurement of 8-OHdG showed they were also not the major source of heterogeneity. Given the above, further studies with uniform standards of detection assay and analysis method to evaluate the expression levels of 8-OHdG are required to elucidate the role of 8-OHdG in human cancers.

## Conclusion

This meta-analysis found that highly expressed 8-OHdG in tumor tissues may be a predictor of prognosis in most solid tumors. However, especially in breast cancer, low 8-OHdG expression is associated with poor prognosis, which is partly because of the increased antioxidant mechanisms in breast cancer tissues. This study demonstrates for the first time that 8-OHdG expression is associated with the prognosis of cancer patients. In the future, whether the expression level of 8-OHdG can be used as a biomarker for the prognosis of all human cancers requires more research.

## Supplementary information

**Additional file 1: Table S1.** Inclusion and exclusion criteria.

## Data Availability

The datasets supporting the conclusions of this article are included within the article.

## Abbreviations

8-OHdG: 8-hydroxy-2′-deoxyguanosine
95% CI: 95% confidence interval
CSS: Cancer-specific survival
DFS: Disease-free survival
dNTP: deoxynucleoside triphosphate
EFS: Event-free survival
ELISA: Enzyme-linked immunosorbent assay
HRs: Hazard ratios
IHC: Immunohistochemistry
LCEC: Liquid chromatography electrochemistry
MTH1: MutT Homolog 1
MUTYH: MutY homolog
NA: Not available
NOS: Newcastle-Ottawa Scale
Nrf2: NF-E2-related factor 2
OGG1: 8-oxoguanine DNA glycosylase
OS: Overall survival
PFS: Progression-free survival
RFS: Recurrence-free survival
ROS: Reactive oxygen species
