# Supplementary material for: Prognostic significance of 8-hydroxy-2′-deoxyguanosine in solid tumors: a meta-analysis
Source: BMC Cancer. 2019 Oct 24;19:997. doi: 10.1186/s12885-019-6189-9 (PMC6813135; doi:10.1186/s12885-019-6189-9)
Supplement: Supplementary file 1 — Additional file 1: Table S1. Inclusion and exclusion criteria. [file 12885_2019_6189_MOESM1_ESM.docx]

| Inclusion criteria | The prognostic values of 8-OHdG in any type of human solid tumors was discussed |
| --- | --- |
|  | All cancer patients were diagnosed according to the gold standard for diagnosis, based on histopathological or histological examinations; |
|  | 8-OHdG levels in tumors, blood samples or urine were estimated in each study |
|  | The patients were divided into two groups according to the levels of 8-OHdG |
|  | Sufficient data should be provided to obtain hazard ratios (HR) for survival rates and their 95% confidence intervals(95%CI) |
| Exclusion criteria | Case reports, letters, editorials, comments, reviews, meta-analysis, expert opinions or any other reviews that contained no original data information, laboratory articles, unpublished articles and conference abstracts |
|  | Full text could not be obtained |
|  | Studies written in non-English |
|  | Duplicated publications |
|  | No survival data or insufficient data to be extracted |
|  | Survival data was acquired based on animal studies and no follow-up was carried out in patients |

Table S1. Inclusion and exclusion criteria

Studies included in this meta-analysis should meet the inclusion and exclusion criteria as above.
